# Dickkopf-1 Is a Biomarker for Systemic Lupus Erythematosus and Active Lupus Nephritis

**DOI:** 10.1155/2017/6861575

**Published:** 2017-03-08

**Authors:** Jing Xue, Jiali Yang, Lijuan Yang, Shaolan Zhou, Chen Ji, Xuemei Wang, Nan Yu, Xiaoming Liu, Shuhong Chi

**Affiliations:** ^1^The General Hospital of Ningxia Medical University, Yinchuan 750004, China; ^2^College of Life Science, Ningxia University, Yinchuan, Ningxia 750021, China; ^3^Department of Rheumatology, The General Hospital of Ningxia Medical University, Yinchuan 750004, China; ^4^Institute of Human Stem Cell Research at The General Hospital of Ningxia Medical University, Yinchuan, Ningxia 750004, China

## Abstract

An early diagnosis of lupus nephritis (LN) has an important clinical implication in guiding treatments of systemic lupus erythematosus (SLE) in clinical settings. In this study, the concentrations of Wnt-3A, Frizzled-8 (FZD-8), and Dickkopf-1 (DKK-1) of Wnt signaling, as well as their diagnostic values for accessing LN, were evaluated by ELISA in sera and urine of 111 SLE patients (31 with LN and 80 without LN) and 70 healthy cohorts. Significantly more abundances of DKK-1 protein were determined in both of sera and urine of SLE patients compared to healthy cohorts (*p* < 0.0001); in particular the serum DKK-1 concentration was even higher in LN-SLE patients relative to non-LN SLE subjects (*p* < 0.0001). Intriguingly, concentrations of above examined proteins in SLE patients showed no correlation between serum and urine. Moreover, a combination of DKK-1 with anti-dsDNA and/or levels of complement C3 and C4 could not increase the specificity and/or sensitivity for identification of patients with LN diseases, but both ROC curve and multiple-factor nonconditional logistic regression analysis showed that serum DKK-1 was considered better positive biomarker for identification of LN in SLE patients. These results imply that serum and/or urine DKK-1 may be a valuable and independent biomarker for identification of SLE patients with LN.

## 1. Introduction

Systemic lupus erythematosus (SLE) is a chronic autoimmune disease that can be characterized by producing various autoantibodies against self-antigens (autoantigens) [1]. The process of SLE pathogenesis can affect multiple systems and major organs, among which lupus nephritis (LN) is one of the most common major organ manifestations and the main cause of morbidity and mortality in SLE patients [2]. In this regard, LN may affect up to 40–80% of SLE patients, partially owing to adverse effects (AEs) of an immunosuppressive treatment for LN on kidney, which may result in chronic renal failure and sequentially increase the morbidity and mortality in SLE patients [1]. This suggests that an involvement of renal disease activity is one of the most important prognostic factors for patients with SLE, and the identification of LN in SLE patients thus has an important clinical implication in guiding the treatment of SLE, which may avoid an immunosuppressive overtreatment in clinical settings [3].

In general, SLE is recognized as a disease that is primarily attributed to autoantibodies, cytokines, and immune complex deposition, and a compelling body of study has demonstrated cytokines such as soluble interleukin-7 receptor (sIL-7R) and autoantibodies to complement C1q, histone, chromatin, and nuclear and double-strand DNA (dsDNA) alone or in combination with anti-C1q, anti-dsDNA, and/or antibodies and/or nucleosome were strongly correlated with renal diseases and could be used for prognosis of patients with LN [2, 4–7]. However, antibodies to dsDNA and the reduction of complements were also found in non-LN patients and clinically inactive SLE patients with a relatively high percentage [8]. Such a lack of specificity of anti-dsDNA antibodies for renal flare was also observed in other conventional parameters such as antinuclear antibody (ANA), levels of complements C3 and C4, proteinuria, and urine sediment [9], which thus led to search other reliable biomarkers for identifying SLE patients with active nephritis [10].

The Wnt signaling has been demonstrated to play crucial roles in several biological aspects, including cellular proliferation, embryonic development, tissue homeostasis, development of immune system, and other systemic effects [11]. In addition to its dispensable roles in the development of T cells and the immune system, mounting evidence has recently suggested that it is involved in the pathogenesis of many types of autoimmune diseases, including rheumatoid arthritis (RA), SLE, spondyloarthritis (PsA), and ankylosing spondylitis (AS) [12–17]. In SLE patients, aberrant Wnt/*β*-catenin signaling was observed in both peripheral B and T cell fractions [18]. Based on the dependence of *β*-catenin, Wnt signaling pathways can be thus further characterized by Wnt/*β*-catenin or canonical pathway and several “noncanonical pathways” (*β*-catenin independent). The latter includes the planer cell polarity (PCP), c-Jun N-terminal protein kinases (JNK), protein kinase C/calcium (PCK/Ca^2+^) pathway, receptor-like tyrosine kinase (RYK), and receptor tyrosine kinase-like orphan receptor (Ror) pathways [11]. Among these, the Wnt/*β*-catenin signaling pathway is the most investigated and the best characterized Wnt signaling pathway.

Activation of Wnt/*β*-catenin signaling can be triggered by the binding of Wnt ligand to its coreceptor low-density lipoprotein receptor-related protein 5 (LRP-5) or LRP6 and frizzled (FZD) family of proteins [19]. Intriguingly, Wnt signaling can also be regulated by extracellular antagonists such as Wise (Sostdc1), secreted frizzled-related protein (SFRP), the Wnt inhibitory factor 1 (WIF1), Cerberus, and the Dickkopf (DKK) family of secreted proteins [20]. Among them, the DKK family of Wnt antagonists has recently spurred increased interests. The DKK family comprises four members of proteins, DKK-1, DKK2, DKK3, and DKK4, which are synthesized as precursor proteins activated by a proteolytic cleavage [21]. The DKK-1 are the most studied members of this family, which can inhibit the Wnt signaling by binding to LRP-5/6 and then degrading the coreceptor and thus have been considered as potential targets in diseases with an aberrant Wnt signaling activity [22, 23].

In RA patients, serum DKK-1 levels were correlated with parathyroid hormone, bone erosions, and bone mineral density [24], and DKK-1 polymorphisms of DKK-1 were associated with the RA structural severity and expression of DKK-1 protein [25]. Activation of DKK-1 and the TNF-alpha-stimulated integrin-related FAK signaling could induce the dissociation of beta-catenin/E-cadherin, which in turn promoted RA fibroblast-like synoviocytes (FLS) migration [26], and such dysregulated DKK-1 pathway could be involved in the pathogenesis and perpetuation of the inflammatory response in early clinically apparent stages of RA [27]. Similarly, in patients with SLE, an aberrant expression of Wnt/*β*-catenin signaling related genes HIG2, TCF7, KHSRP, WWP1, SMAD3, TLK2, AES, CCNI, and PIM2 was observed in the peripheral blood CD4^+^ T cells in patients with SLE [18, 28]. These genes have been demonstrated to play an important role in the regulation of T cell proliferation and differentiation [29]. Both human and LN MRL/lpr mouse studies indicated that the DKK-1 protein was significantly higher in the sera of SLE patients compared with control subjects, and the LN MRL/lpr mice exhibited a phenotype with an enhanced Wnt/*β*-catenin activity, accompanied by an increased level of DKK-1 in the renal tissues and sera and an increased frequency of apoptotic cells of the renal tubular and renal interstitial tissues [30]. Notably, the beta-catenin transcriptional activity in leukocytes of lupus-prone mice and SLE patients was diminished, particularly in myeloid cells [31]. Such an activated Wnt signaling was further evidenced in human renal tissues of patients with LN by accessing *β*-catenin at both transcriptional and translational levels using assays including immunohistochemistry staining, qRT-PCR, and western blotting, suggesting that a dysregulated Wnt/*β*-catenin signaling was related to the pathogenesis of LN and might play a role in the renal fibrosis [15].

Recently, an increasing number of studies have demonstrated the potentially diagnostic and/or prognostic values of DKK-1 in varied cancers, such as lung cancer [32–34], gastrointestinal cancers [23], pancreatic cancer [35], and hepatocellular carcinoma [36–38], as well as rheumatic disorders, including RA [24] and AS [39, 40]. Together with aforementioned pathogenic roles of Wnt signaling in LN development, these studies clearly imply that Wnt signaling, in particular the DKK-1, may be a novel biomarker for identification of LN for patients with SLE. To this end, we evaluated concentrations of Wnt-3A, a ligand of Wnt/*β*-catenin signaling, FZD-8, a receptor of the signaling, and DKK-1 in the sera and urine of 111 SLE patients in a single center and investigated the clinical significance of such antibodies alone or in combination with anti-dsDNA antibodies and/or serum levels of C3 and C4 for accessing active nephritis in SLE patients in the present study. Our results showed that only DKK-1 protein was increased in both sera and urine of SLE patients, but only the serum DKK-1 exhibited a statistical difference between LN-SLE patients and non-LN-SLE subjects. However, a combination of DKK-1 with anti-dsDNA and/or levels of complements C3 and C4 could not increase the specificity and/or sensitivity for identification of patients with LN diseases. These data suggest that the serum and/or urine DKK-1 may be a valuable and independent biomarker for identification of SLE patients with active LN.

## 2. Materials and Methods

### 2.1. Ethics Statement

Human blood and urine samples were collected with a protocol approved by the Ethic Committee for the Conduct of Human Research at Ningxia Medical University (NXMU-E2012-102p). Written consent was obtained from every individual according to the Ethic Committee for the Conduct of Human Research protocol. For the participants younger than 18 years old, written informed consent was obtained from their guardians or parents on behalf of the children. No special informed consent was required for Chinese Hui, Man, and Mongolian minorities in this study. All participants provided a written informed consent for the publication of the data. The PI of this study maintains human research records, including signed and dated consent documents, for ten (10) years after the age of majority. The Ethic Committee the Conduct of Human Research at Ningxia Medical University approved the consent procedure for this study (NXMU-2012-102e).

### 2.2. Blood and Urine Samples

Blood and urine samples of 111 consecutive SLE patients (103 females and 8 males) were collected from the outpatient rheumatology clinics of the General Hospital of Ningxia Medical University from January to December 2015. The mean ± SD age for the SLE patients at the time of the sample drawn was 38.23 ± 11.17 years old (range 17 to 76), with an average duration of diseases of 6.14 ± 4.24 (0.3 to 14 years). The American College of Rheumatology (ACR) criteria were used to diagnose a patient with SLE [41, 42], and the disease activity was defined according to SLE Disease Activity Index (SLEDAI) criteria [43, 44]. A patient with SLEDAI ≥ 10 was defined as active SLE. Renal involvement was defined based on clinical and laboratory manifestations. An active LN was defined as urine protein excretion ≥500 mg/day or cellular casts [41]. Sera and urine of 70 gender and age-matched healthy individuals (7 males and 63 females) were also collected. These healthy control cohorts were recruited from those who had undergone comprehensive medical screening at the General Hospital of Ningxia Medical University and who had no history of chronic diseases, no family history of autoimmune diseases. The demographics of individuals involved in this study were outlined in Table 1. All sera were treated with heparin. Both serum and urine samples were frozen in 100 *μ*L aliquots at −80°C until analyzed. The ethnic populations of subjects in this study included Chinese Han, Chinese Hui, Chinese Man, and Chinese Mongolian Mongolian. Ethnic populations were determined based on criteria of purely Chinese Han, Hui, Man, or Mongolian descents for at least three generations (Table 1). There was no genetic relationship among these individuals. All the samples were collected under informed consent.

### 2.3. Detection of Wnt-3A, FZD-8, and DKK-1 by Enzyme-Linked Immunosorbent Assay (ELISA)

Concentrations of Wnt signaling Wnt-3A, FZD-8, and DKK-1 proteins in serum and urine were measured by ELISA using commercially available kits according to the manufacturer's instructions. The ELISA Kits for human Wnt-3A and FZD-8 were products of R&D Systems China Co., Ltd. (Shanghai, China); the ELISA Kit for DKK-1 was a product of BosterBio Inc. (Wuhan, China). For measurement of Wnt-3A and FZD-8, both urine and serum were diluted with dilution buffer by 5. For detection of DKK-1 protein, the serum and urine were diluted with dilution buffer by 20 and 10, respectively. Briefly, diluted samples were added to each well; the wells were then washed with high ionic strength buffer after being incubated at room temperature for 1 h. Then horseradish peroxidase-conjugated anti-human IgG supplied with the kit was used as the secondary antibody. After 30 min incubation, the wells were extensively washed for three times, followed by the addition of 100 *μ*l trimethylbenzene solution and incubation for 30 min before an 100 *μ*l of stopping solution was added to each well. The optical density was then measured at 450 nm. The absorbance (OD_450 nm_) was then converted into a concentration (ng/mL) through standard curve. Other laboratory data, including urinalysis, serum levels of complements C3 and C4 and hemoglobin, antinuclear antibodies (ANA), anti-dsDNA antibodies, antiribonucleoprotein, perinuclear antineutrophil cytoplasmic antibody (pANCA), antibodies to Sjogren's syndrome A (SSA) and B (SSB), and anti-Smith (Sm) were also recorded, respectively. The sensitivity, specificity, and predictive values were calculated using formula described in a previous report [45].

### 2.4. Statistical Analysis

All laboratory data were entered into and extracted from PRISM (version 5) (GraphPad Software, La Jolla, CA, USA) and/or SPSS for Windows (version 17.0) (SPSS Inc., Chicago, IL, USA). Statistical evaluation of the data was performed by one-way ANOVA when more than two groups were compared with a single control and *t*-test for comparison of differences between the two groups. ROC (receiver operator characteristic) curve was used to find out the best cut-off value and validity of certain variable. The multiple-factor nonconditional logistic regression analysis was employed with SPSS Software. The association between qualitative variables was evaluated by Spearman correlation. Data was presented as the mean standard error of mean (SEM) or mean ± standard deviation (SD). A *p* value of less than 0.05 was considered statistically significant. ^*∗*^*p* < 0.05; ^*∗∗*^*p* < 0.01; and ^*∗∗∗*^*p* < 0.0001.

## 3. Results

### 3.1. SLE Demographics Data

The unselected SLE population studied in this study included 103 (92.8%) females and 8 males (7.2%) with a mean age of 38.23 ± 11.17 years old (range 17 to 76), and the average duration of diseases was 6.14 ± 4.24 (0.3 to 14 years) at the time of the sample collection (mean ± SD). The mean of SLEDAI score of SLE was 8.55 ± 5.14 (range 0 to 27). The majority of distribution of ethnic population was 80.2% of Chinese Han (Table 1). The demographic data of LN-SLE and non-SLE patients, as well as control healthy cohorts, were presented in Table 1. Laboratory parameters between active and inactive SLE, with and without renal involvement were also listed in Table 2.

### 3.2. Concentrations of Wnt-3A, FZD-8, and DKK-1 Proteins in Sera of SLE Patients

**Figure 1:**
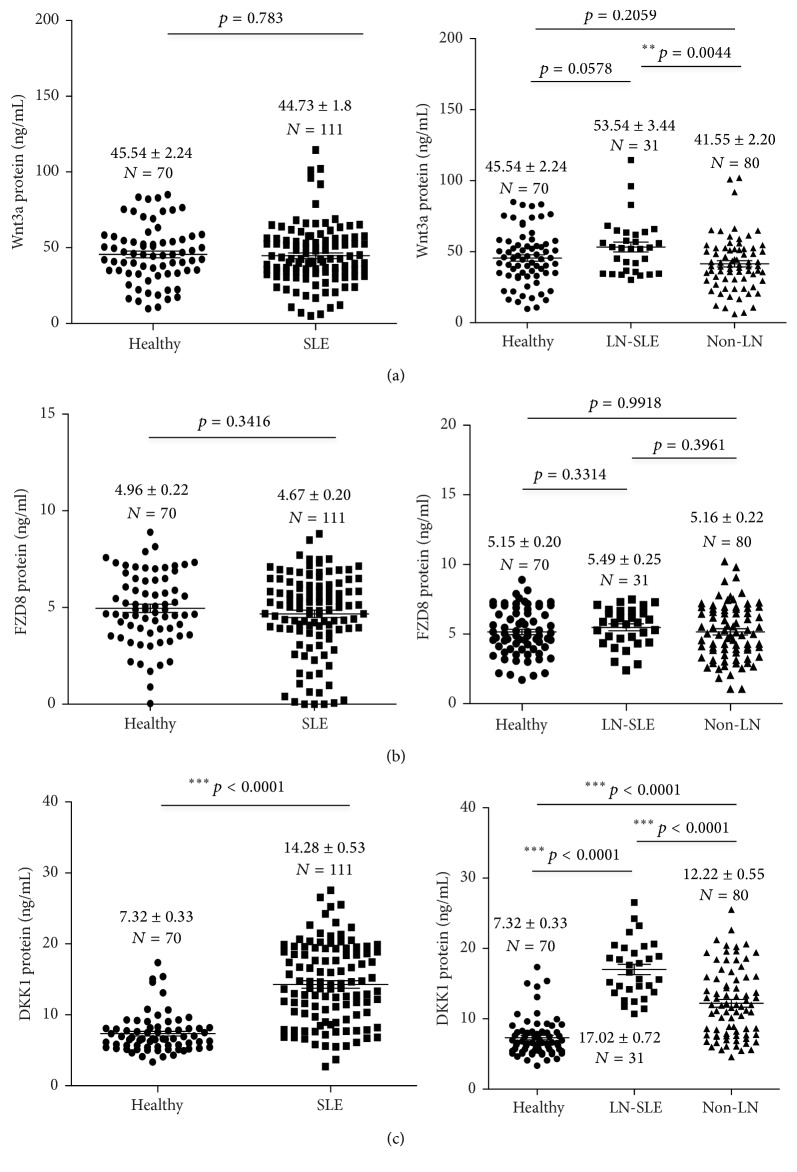
The serum concentrations of Wnt-3A, FZD-8, and DKK-1 proteins in healthy individuals and SLE patients. (a) The concentration of serum Wnt-3A protein in healthy individuals and SLE patients. No statistical difference was determined between healthy individuals and SLE patients (left panel), but more abundant Wnt-3A protein was found in sera of SLE patients with renal involvement (LN-SLE) compared with non-LN-SLE patients (right panel, *p* = 0.0044). (b) The concentration of serum FZD-8 protein in healthy individuals and SLE patients. No statistical difference was determined between healthy individuals and SLE patients (left panel), and LN-SLE and non-LN-SLE patients (right panel). (c) The concentration of serum DKK-1 protein in healthy individuals and SLE patients. Statistical differences were found between healthy individuals and SLE patients (left panel, *p* < 0.0001), LN-SLE (*p* < 0.0001) and non-LN-SLE patients (right panel, *p* < 0.0001). More abundant serum DKK-1 protein was detected in LN-SLE patients relative to healthy individuals and SLE patients without renal flare, and the highest level of serum DKK-1 protein was determined in LN-SLE patients. Bars indicate the average levels of indicated proteins in each group. Compared with the respective healthy and non-LN groups, ^*∗∗*^*p* < 0.01 and ^*∗∗∗*^*p* < 0.001. Data presents as the mean ± SEM in each groups.

In order to determine whether Wnt signaling was correlated with SLE activity, serum concentrations of Wnt-3A, FZD-8, and DKK-1 were evaluated in SLE patients with and without renal flare and healthy subjects. Serum concentrations of respective Wnt-3A, FZD-8, and DKK-1 were 45.54 ± 2.24, 4.96 ± 0.22, and 7.32 ± 0.33 ng/mL for healthy subjects; 44.73 ± 1.86, 4.67 ± 0.20, and 14.28 ± 0.53 ng/mL for SLE patients; 53.54 ± 3.44, 5.49 ± 0.25, and 17.02 ± 0.72 ng/mL for LN-SLE patients; and 41.55 ± 2.20, 5.16 ± 0.22, and 12.22 ± 0.55 ng/mL for non-LN patients (Table 3 and Figure 1). Surprisingly, no significant difference of abundance of serum Wnt-3A protein was found between SLE patients and healthy subjects, although a statistical higher level of Wnt-3A was determined in LN-SLE patients relative to non-LN-SLE patients (Figure 1(a)). Interestingly, there was no significant difference of serum FZD-8 protein detected between healthy individuals and SLE patients and between SLE patients with LN and without LN (Figure 1(b)). Notably, a significantly more abundant serum DKK-1 protein was determined in SLE patients compared to healthy cohorts (*p* < 0.0001) (Figure 1(c)). More importantly, a strikingly higher level of serum DKK-1 was found in SLE patients with renal involvement in comparison with those without renal flare (*p* < 0.0001) (Figure 1(c)).

### 3.3. Concentrations of Wnt-3A, FZD-8, and DKK-1 Proteins in Urine of SLE Patients

**Figure 2:**
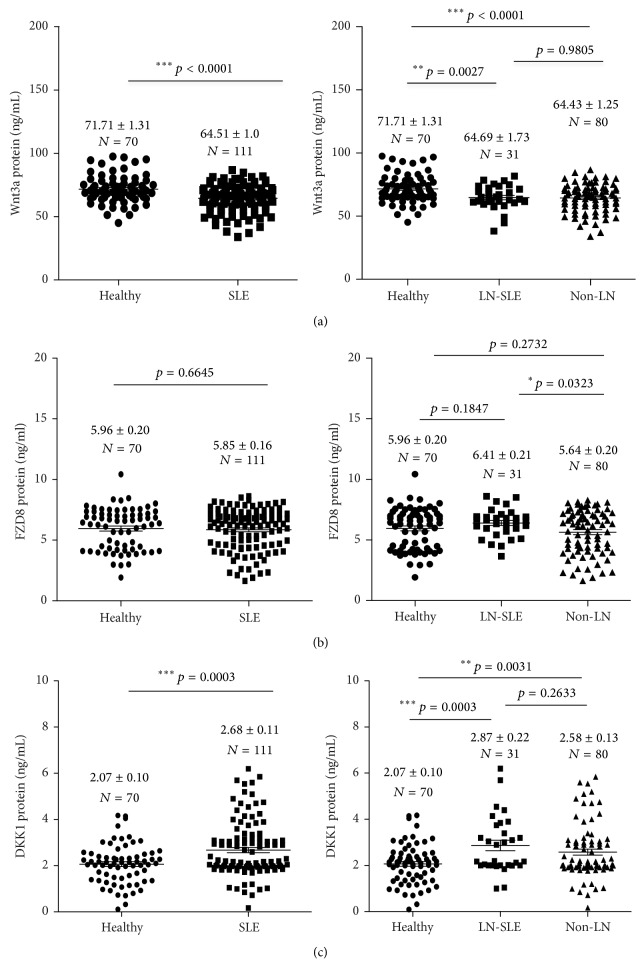
The concentrations of Wnt-3A, FZD-8, and DKK-1 proteins in urine of healthy individuals and SLE patients. (a) The concentration of urine Wnt-3A protein in healthy subjects and SLE patients. No statistical difference was determined between healthy individuals and SLE patients (left panel) and LN-SLE and non-LN-SLE patients as well (right panel). (b) The concentration of urine FZD-8 protein in healthy individuals and SLE patients. No statistical difference was determined between healthy individuals and SLE patients (left panel), but a moderately more abundant FZD-8 protein was found in the urine of LN-SLE patients compared with SLE patients without renal involvement (right panel, *p* < 0.05). (c) The concentration of urine DKK-1 protein in healthy individuals and SLE patients. A statistical difference was found between healthy individuals and SLE patients (left panel, *p* < 0.0001) but not between LN-SLE and non-LN-SLE patients (right panel, *p* = 0.2633). Bars indicate the average levels of indicated proteins in each group. Compared with respective healthy and non-LN groups, ^*∗*^*p* < 0.05, ^*∗∗*^*p* < 0.01  ^*∗∗∗*^*p* < 0.001. Data presents as the mean ± SD in each groups.

Since an activated Wnt signaling was reported in kidney of SLE patients with renal involvement, we next evaluated levels of Wnt-3A, FZD-8, and DKK-1 in urine of SLE patients. Urine levels of respective Wnt-3A, FZD-8, and DKK-1 were 71.71 ± 1.31, 5.96 ± 0.20, and 2.07 ± 0.10 ng/mL for healthy individuals; 64.51 ± 1.01, 5.85 ± 0.16, and 2.68 ± 0.11 ng/mL for SLE patients; 64.69 ± 1.73, 6.41 ± 0.21, and 2.87 ± 0.22 ng/mL for LN-SLE patients; and 64.43 ± 1.25, 5.64 ± 0.20, and 2.58 ± 0.13 ng/mL for non-LN patients (Table 3 and Figure 2). Of note, a significantly less abundant Wnt-3A protein could be detected in urine of SLE patients relative to control subjects (*p* < 0.0001), but no difference was found between SLE patients with and without renal flare (*p* = 0.9805) (Figure 2(a)). Similar to serum FZD-8, there was no statistical difference found in urine FZD-8 between healthy individuals and SLE patients, despite a moderately more abundance of urine FZD-8 protein was determined in SLE patients with LN relative to those without renal involvement (*p* < 0.05) (Figure 2(b)). Of importance, a significantly higher level of urine DKK-1 protein was determined in SLE patients relative to healthy individuals (*p* = 0.0003) (Figure 2(c)), but unlike what is seen in serum, no difference of the abundance of urine DKK-1 was detected between SLE patients with renal involvement and those without LN (*p* = 0.2633) (Figure 2(c)).

### 3.4. Correlations of Wnt-3A, FZD-8, and DKK-1 Concentrations in Sera and Those in Urine of SLE Patients

**Figure 3:**
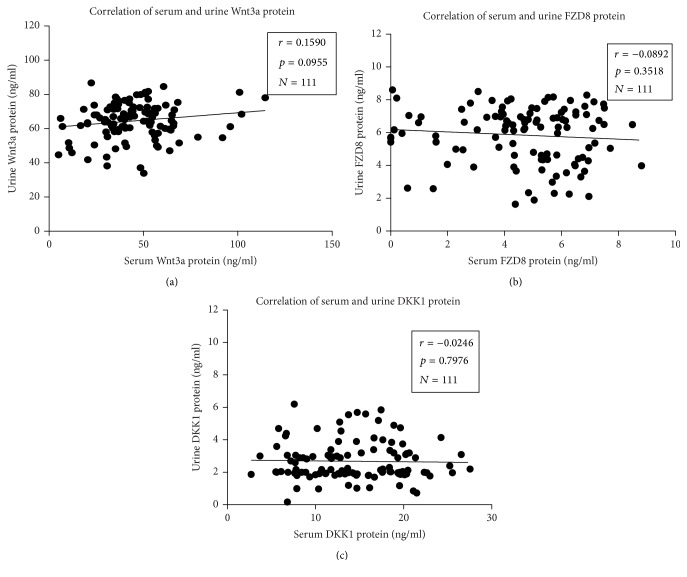
Correlations between serum and urine concentrations of Wnt-3A, FZD-8, and DKK-1. (a) Correlation between Wnt-3A protein levels in serum and urine. (b) Correlation between FZD-8 protein levels in serum and urine. (c) Correlation between DKK-1 protein levels in serum and urine. No significant correlation was determined between the serum and urine for all the three tested proteins. Spearman *r* and *p* values are displayed on each graph. A *p* value was determined by the two-tailed Pearson correlation test.

Above data showed DKK-1 protein was more abundant in both serum and urine of SLE patients compared with those of healthy subjects; the correlation of above examined proteins in serum and urine was analyzed. Unexpectedly, there was no association between serum and urine in SLE patients determined for Wnt-3A (*r* = 0.159, *p* = 0.0955, and *N* = 111), FZD-8 (*r* = −0.0892, *p* = 0.3518, and *N* = 111), and DKK-1 (*r* = −0.0246, *p* = 0.7976, and *N* = 111) (Figure 3). In addition, association analysis serum or urine DKK-1 and clinical SLEDAI score or other serological biomarkers including anti-C1q, anti-dsDNA, ANA, and C3 and C4 levels also showed no significant correlation between DKK-1 and aforementioned serological biomarkers or SLEDAI score (data not shown).

### 3.5. Significance of DKK-1 for the Identification of Patients with LN

**Figure 4:**
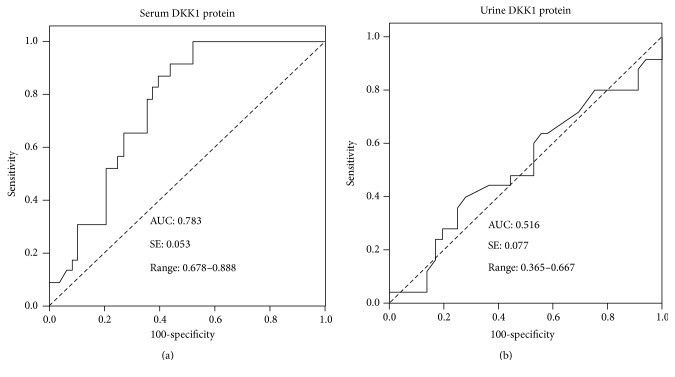
ROC curve for DKK-1 in active lupus nephritis. (a) ROC curve for serum DKK-1 in active lupus nephritis. (b) ROC curve for urine DKK-1 in active lupus nephritis.

Higher levels of serum and urine DKK-1 protein were detected in SLE patients compared with healthy subjects; in particular serum DKK-1 was even more abundant in patients with LN-SLE in comparison with non-LN-SLE patients (Table 3, Figures 1 and 2). In order to evaluate the significance of serum DKK-1 in clinical settings, we analyzed the sensitivities and specificities of serum DKK-1, anti-dsDNA antibodies, and levels of C3 and C4 alone or in a combination for the identification of patients with LN (Table 4). DKK-1 alone displayed a superior sensitivity for identifying patients with LN to serum levels of C3 and C4 but inferior to anti-dsDNA antibodies (Table 4). Furthermore, a combination of serum DKK-1 and anti-dsDNA antibodies or serum levels of C3 and C4 could not increase specificities and sensitivities in identification of patients with LN in comparison with these serological markers alone (Table 4). Of interest, the multiple-factor nonconditional logistic regression analysis of impacts on LN-SLE suggested that serum DKK-1 was a factor with clinical significance for LN-SLE with an odd ratio (OR) (95% CI) of 1.271 (*p* = 0.045) (Table 5). The ROC curve also showed that DKK-1 (Figure 4), particularly the serum DKK-1, was considered better positive biomarker than negative in LN with higher sensitivity (Figure 4). The area under curve (AUC) for serum DKK-1 was 0.783 (SE: 0.053; range: 0.678–0.888) (Figure 4(a)); and the AUC for urine DKK-1 was 0.516 (SE: 0.077; range: 0.365–0.667). These results may imply that the serum DKK-1 protein may be an independent biomarker for identification of LN in SLE patients.

## 4. Discussion

With increasing appreciation for findings that dysregulated Wnt/*β*-catenin signaling is involved in the development and pathogenesis of many disease types including cancers and autoimmune disorders, such as the SLE [16, 46, 47], several key molecules in Wnt/*β*-catenin signaling cascade have been investigated as biomarkers for disease diagnosis and/or prognosis [48–53], among them Wnt signaling inhibitor DKK-1 has spurred an increased interest in both cancers and autoimmune diseases [24, 27, 30, 34, 37, 40, 54–56]. In this report, we examined concentrations of Wnt-3A, FZD-8, and DKK-1 in the serum and urine of SLE patients and analyzed their diagnostic value for identification of SLE patients with renal flare. The results showed that significant more abundances of DKK-1 protein were determined in both sera and urine of SLE patients compared with healthy cohorts (*p* < 0.0001); in particular the serum DKK-1 concentration was even higher in LN-SLE patients relative to non-LN-SLE subjects (*p* < 0.0001). Consistently, less abundant Wnt-3A was also determined in urine of SLE patients relative to healthy cohorts, although there was no difference of Wnt-3A observed between sera of these two groups. In contrast, there was no significant difference of FZD-8 found in neither sera nor urine in this study. Intriguingly, the concentrations of above examined proteins in sera and urine showed no correlation. Moreover, a combination of DKK-1 with anti-dsDNA and/or levels of complements C3 and C4 could not increase the specificity and/or sensitivity for identification of patients with LN diseases, but both the ROC curve and the multiple-factor nonconditional logistic regression analysis showed that the serum DKK-1 was considered better positive biomarker for identification of LN in SLE patients. These results imply that the serum and/or urine DKK-1 may be valuable and independent biomarker for identification of SLE patients with active LN. These data are in line with previous findings of DKK-1 and Wnt signaling in SLE patients [15, 30, 56].

Several lines of evidences have demonstrated that aberrant canonical Wnt/*β*-catenin signaling is involved in autoimmune disorders. For instance, Wnt signaling has been recognized to play a central role in the bone development and homeostasis in adulthood, and a dysregulation of this signaling is associated with bone pathologies [24, 34]. In this context, Dickkopf-1 (DKK-1) is required for embryonic head development, which has been implicated in osteoclast dysregulation in RA [56, 57]. A blockade of DKK-1 may thus serve to restore the osteoblast-osteoclast balance and repair bone erosion in RA joints. Indeed, an evoked serumDKK-1 level was determined in RA patients as compared with healthy individuals, which was also found to correlate with parathyroid hormone, bone erosions, and osteoporosis [24]. In addition, several preclinical studies further showed that a neutralizing DKK-1 and/or enhancing Wnt/*β*-catenin signaling could be an effective therapeutic option in treatment of bone pathologies [24, 57–59]. Similarly, Wnt signaling inhibitors sclerostin and DKK-1 have been investigated as biomarkers for disease activity in ankylosing spondylitis (AS), and a lower level of serum DKK-1 was observed in AS patients [39, 60]. Interestingly, the amount of DKK-1 protein was found not to be consistently correlated to its capacity of binding to the LRP coreceptor in sera of AS patients, in which the DKK-1 in the sera was less able to suppress *β*-catenin translocation to the nucleus than control sera, implying that the DKK-1 might be dysfunctional in AS patients [61]. Mechanistically, aberrant TNF was suggested to contribute to inducing the evoked expression of DKK-1 and sclerostin in RA, and a neutralization of DKK-1 with antibodies led a reversed phenotype of erosions in several inflammatory arthritis murine models and altered the phenotype from bony erosion to proliferation [62]. Such an elevated serum DKK-1 level was also reported in SLE patients with bone erosion [30]. Furthermore, the blockade of DKK-1 with this antibody could promote the fusion of the sacroiliac joints in TNF-engineered RA mouse model [63]. Mechanistically, the TNF-induced release of DKK-1 might be able to inhibit Wnt signaling, which in turn diminished osteoprotegerin (OPG) expression and osteoblastogenesis and increased osteoclast activity and erosion [62]. This notion was in accordance with the finding of a decreased serum DKK-1 in RA patients but not in the AS patients treated with TNK inhibitors [56, 62]. These findings suggest that Wnt signaling, particularly the DKK-1, is a biomarker for autoimmune disease diagnosis and a target for disease treatment. Indeed, a recent meta-analysis of seven case-control trials with a total of 300 AS patients, 136 RA patients, and 232 healthy controls found that serum levels of DKK-1 were significantly higher in AS patients relative to normal controls, although there was significant difference in DKK-1 serum levels observed between RA patients and healthy controls [56].

With respect to SLE, dysregulated Wnt signaling activity was first determined in sera and kidneys of mice during lupus development by gene expression analysis [13]. In this report, an increased canonical Wnt/*β*-catenin signaling activity was determined in kidneys of (NZB × NZW) F1 mice during progression of lupus nephritis, which was paralleled by an increase in renal and serum levels of DKK-1. Notably, sera collected from mice with proteinuric stage of LN showed strong Wnt inhibitory effects, and the concentration of DKK-1 was comparable to those observed in lupus-prone mice induced apoptosis in tubular and mesangial cells in vitro [13]. This study thus indicated that Wnt signaling activity was enhanced in the kidney with LN, which was accompanied by increased renal and serum DKK-1 levels, suggesting that the canonical Wnt signaling was involved in the turnover of extracellular matrix constituents and represents a potential mediator of the morphologic changes that occur within the glomerulus during the development of nephritis, and the DKK-1 might be pivotal element in the development and progression of systemic and end-organ disease manifestations in SLE [13]. Consistent with findings in this SLE mouse model, Wang et al. recently reported enhanced activation of Wnt/*β*-catenin signaling in SLE patients with LN [15]. In this study, the authors evaluated the expressions of *β*-catenin, DKK-1, and AXIN-2 mRNAs and proteins in the renal biopsy of patients with LN-SLE by a quantitative RT-PCR and immunohistochemistry assay, respectively; the concentration of plasma DDK1 was also measured by ELISA. The immunohistochemistry and western blotting showed an increased expression of *β*-catenin in the renal tissues of patients with LN-SLE compared with control samples, and more abundant *β*-catenin and AXIN-2 transcripts were also detected in the LN renal tissues relative to controls. Of note, such an increased abundance of *β*-catenin transcript was positively correlated with the creatinine clearance rate (Ccr) and negatively correlated with chronicity indices of renal tissue injury [15]. More importantly, an evoked concentration of DKK-1 protein was found in the plasma of LN patients in comparison with controls, which was negatively correlated with anti-dsDNA antibody level and positively with serum C3 concentration [15].

In agreement with above findings, an increased concentration of DKK-1 was determined in the serum of SLE patients compared with healthy subjects. Even more notably, a significantly higher level of serum DKK-1 was detected in LN-SLE patients relative to those with non-LN-SLE. However, no statistical difference between SLE patients and control individuals was determined for Wnt-3A and FZD-8 in sera. As a manifestation with renal involvement in LN patients, concentrations of Wnt-3A, FZD-8, and DKK-1 were also examined in our study. Interestingly, a statistically less abundant Wnt-3A and more abundant DKK-1 were found in urine of SLE patients as compared with healthy subjects, although no significant difference of urine DKK-1 level was determined between patients with LN-SLE and non-LN-SLE. Of note, no correlation for respective concentrations of Wnt-3A, FZD-8, and DKK-1 was determined between sera and urine in SLE patients. In disagreement with results reported by Wang and colleagues [15], no correlation between concentration of serum or urine DKK-1 and other serological biomarkers, such as anti-dsDNA antibody and C3 and C4 levels, was determined. In our study, serum sample but not plasma was employed for examinations, and the ELISA Kit for DKK-1 measurement was also different from the study by Wang et al. [15]. These variations may be part of the reason that caused the discrepancy between these two studies, which required further investigation. Of importance, both the ROC curve and the multiple-factor nonconditional logistic regression analysis showed that the serum DKK-1 was considered better positive biomarker for identification of LN in SLE patients. These data may imply that DKK-1 is an independent biomarker for identification of LN-SLE patients.

## 5. Conclusion

In conclusion, this study in 111 SLE patients confirms previous findings that circulating DKK-1 is a valuable biomarker for identification of SLE patients with LN. Intriguingly, an increased DKK-1 concentration along with a decreased Wnt-3A level was observed in urine of SLE patients relative to healthy cohorts. This finding further supports the notion that Wnt/*β*-catenin signaling pathway plays a key role in the initiation and progression of LN. However, no clinical significance was observed for serum Wnt-3A between SLE patients and control subjects. Interestingly, no correlation between sera and urine of SLE patients was determined for respective Wnt-3A, FZD-8, and DKK-1. In addition, a combination of DKK-1 with anti-dsDNA and/or levels of complements C3 and C4 could not increase the specificity and/or sensitivity for identification of patients with LN, although both the ROC curve and a logistic regression analysis demonstrated that the serum DKK-1 could be considered better positive biomarker. These results thus imply that DKK-1 is an independent biomarker for identification of LN in SLE patients, which warrants for further investigation in clinical settings. Limitations of this study include that only a small number of 111 SLE samples were studied, follow-up data were also lacking, and the LN activity was mainly determined by laboratory parameters and clinical manifestations rather than by pathogenic analysis in renal biopsies. Therefore, this finding deserves confirmation in a larger and more selected population in future.

## Figures and Tables

**Table 1:** Demographics of patients with systemic lupus erythematosus (SLE) without lupus nephritis (LN) (non-LN-SLE) (*N* = 80), SLE with LN (LN-SLE) (*N* = 31), and healthy control cohorts.

Demographics	Non-LN-SLE (*n* = 80)	LN-SLE (*n* = 31)	Healthy (*n* = 70)
Ethnics (Chinese Han*/*Hui*/*Man/Mongolian)	80 (66/12/1/1)	31 (23/8/0/0)	70 (43/27/0/0)
Age (mean ± SD) (range, years old)	40.14 ± 11.90 (18–76)	33.30 ± 9.29 (17–52)	30.14 ± 8.43 (20–65)
Gender (male/female) (% female)	4/76 (95.6)	4/27 (87.1)	7/63 (90.0)
Disease duration (mean ± SD) (range, years)	6.23 ± 4.21 (0.3–12)	5.89 ± 4.32 (0.6–14)	NA
SLEDAI score (range)	7.31 ± 4.83 (0–19)	11.74 ± 5.93 (4–27)	NA

Data represented the mean ± SD analyzed by Student's *t*-test using SPSS. NA: unrelated.

**Table 2:** Association of the presence of laboratory parameters between active and inactive SLE, with and without renal involvement (mean ± SD).

	Activity of SLE			Renal involvement of SLE		
	Active SLE (*n* = 20)	Inactive SLE (*n* = 91)	*p*	LN (*n* = 31)	Non-LN (*n* = 80)	*p*
CANCA (+) number (%)	0/20 (0)	0/91 (0)	NA	0/31 (0)	0/80 (0)	NA
ANA (+) number (%)	17/20 (85.0)	55/91 (86.0)	NA	28/31 (90.3)	44/80 (55.0)	NA
Anti-Rib-P (+) number (%)	2/20 (10.0)	COPD11/91 (12.1)	NA	4/31 (12.9)	9/80 (11.2)	NA
Anti-SSA Ab (+) number (%)	8/20 (40.0)	30/91 (33.0)	NA	15/31 (48.4)	23/80 (28.8)	NA
Anti-SSB Ab (+) number (%)	4/20 (20.0)	11/91 (12.1)	NA	6/31 (19.4)	9/80 (11.3)	NA
pANCA (+) number (%)	1/20 (5.0)	1/91 (1.1)	NA	0/31 (0)	2/80 (2.5)	NA
U1RNP (+) number (%)	9/20 (45.0)	18/91 (19.8)	NA	10/31 (32.3)	17/80 (21.2)	NA
Anti-Smith (+) number (%)	6/20 (30.0)	6/91 (7.0)	NA	4/31 (12.9)	8/80 (10.0)	NA
ACA (+) number (%)	15/20 (75.0)	30/91 (32.4)	NA	20/31 (64.5)	25/80 (31.3)	NA
C3 (*µ*g/mL)	0.48 ± 0.18	0.60 ± 0.19	0.000	0.43 ± 0.18	0.58 ± 0.21	0.0067
C4 (*µ*g/mL)	0.054 ± 0.023	0.57 ± 0.022	0.146	0.055 ± 0.021	0.088 ± 0.026	0.0913
Anti-dsNDA (IU/mL)	214.45 ± 103.76	90.30 ± 70.17	0.000	229.70 ± 111.05	156.91 ± 102.30	0.045

A SLEDAI score not less than 10 was defined as active SLE. Data represented the mean ± SD; variance was analyzed by independent-samples *t*-test using GraphPad Prism5. Ab: antibody; ACL: anticardiolipin; ANA: antinuclear antibody; LN: lupus nephritis; pANCA: perinuclear antineutrophil cytoplasmic antibody; Rib-P: ribosomal P-proteins; RNP: ribonucleoprotein; SSA: Sjogren's syndrome A; and SSB: anti-Sjogren's syndrome B.

**Table 3:** The abundances of Wnt-3A, FZD-8, and DKK-1 proteins in the sera and urine of patients with SLE.

	Serum SLE			Urine SLE		
	Non-LN-SLE (*n* = 80)	LN-SLE (*n* = 31)	*p*	Non-LN-SLE (*n* = 80)	LN-SLE (*n* = 31)	*p*
Wnt-3A	41.55 ± 2.20 (ng/mL)	53.54 ± 3.44	0.0044_ _^*∗∗*^	64.43 ± 1.25	64.69 ± 1.25	0.9805
FZD-8	5.16 ± 0.22 (ng/mL)	5.49 ± 0.25	0.3961	5.64 ± 0.20	6.41 ± 0.21	0.0323_ _^*∗*^
DKK-1	12.22 ± 0.55 (ng/mL)	17.02 ± 0.72	0.000_ _^*∗∗∗*^	2.58 ± 0.13	2.87 ± 0.22	0.2633

Data represented the mean ± SEM; variance was analyzed by independent-samples *t*-test using GraphPad Prism5. ^*∗*^*p* < 0.05; ^*∗∗*^*p* < 0.01; and ^*∗∗∗*^*p* < 0.001.

**Table 4:** Significant difference in levels of serum DKK-1 and anti-dsDNA antibodies, C3, and C4 in patients with LN-SLE compared to non-LN-SLE individuals.

	Sensitivity (%)	Specificity (%)	PPV (%)	NPV (%)
DKK-1 (serum)	24/31 (77.4)	34/80 (42.5)	24/70 (34.3)	46/53 (86.8)
Anti-dsDNA Ab	25/31 (80.6)	45/80 (62.5)	25/60 (41.7)	35/41 (85.4)
Levels of C3 and C4	15/31 (48.4)	40/80 (50.0)	15/55 (27.3)	40/56 (71.4)
Anti-dsDNA Ab, levels of C3 and C4	13/31 (41.9)	39/80 (48.8)	13/54 (24.1)	41/59 (69.5)
DKK-1 (serum) and dsDNA	12/31 (38.7)	33/80 (41.2)	12/59 (20.3)	47/66 (66.7)
DKK-1 (serum), levels of C3 and C4	11/31 (35.5)	33/80 (41.3)	11/58 (19.0)	47/67 (70.1)
DKK-1 (serum) and dsDNA, levels of C3 and C4	11/31 (35.5)	32/80 (40.0)	11/59 (18.6)	48/68 (70.6)

**Table 5:** Multiple-factor nonconditional logistic regression analysis of the impact of serological factors on LN-SLE.

	DKK-1	SLEDAI	Anti-dsDNA	C3	C4	Sm	ANCA
WALD	4.016	1.672	1.818	1.379	0.43	3.332	0.723
*p*	0.045_ _^*∗*^	0.196	0.178	0.24	0.837	0.523	0.465
OR	1.271	1.166	1.008	0.019	0.1	0.1	0.16

^*∗*^ *p* < 0.05; ^*∗∗*^*p* < 0.01; and ^*∗∗∗*^*p* < 0.001.

## Abbreviations

ACR: American College of Rheumatology
ANA: Antinuclear antibody
AS: Ankylosing spondylitis
AUC: Area under the curve
BD: Behcet's disease
BM-MSCT: Bone marrow-mesenchymal stem cell transplantation
C1q: Complement lq
CD: Crohn's disease
CIA: Collagen-induced arthritis
CTL: Cytotoxic T lymphocytes
DKK-1: Dickkopf-1
DM: Dermatomyositis
dsDNA: Double stranded DNA
ILs: Interleukins
LN: Lupus nephritis
JRA: Juvenile idiopathic arthritis
MCTD: Mixed connective tissue disease
MS: Multiple sclerosis
LRP: Low-density lipoprotein receptor-related protein
MSCs: Mesenchymal stem cells
NF-*κ*B: Nuclear factor-kappa B
pANCA: Perinuclear antineutrophil cytoplasmic antibody
pSS: Primary Sjogren's syndrome
RA: Rheumatoid arthritis
RANKL: Receptor activator of nuclear factor-kappa B ligand
RhoA: Ras homolog gene family, member A
Rib-P: Ribosomal P-proteins
RMG: Retinal Müller glial cells
RNP: Ribonucleoprotein
ROC: Receiver operating characteristic
sIL-7R: Soluble interleukin-7 receptor
SFMCs: Synovial fluid mononuclear cells
SFRP4: Frizzled-related protein 4
SLE: Systemic lupus erythematosus
SLEDAI: SLE Disease Activity Index
SSA: Sjogren's syndrome A
SSB: Sjogren's syndrome B
SSc: Systemic sclerosis
TGF-*β*: Transforming growth factor *β*
T1DM: Type 1 diabetes mellitus
TNF-*α*: Tumor necrosis factor alpha
WISP3: Wnt1-inducible signaling pathway protein 3.

## References

[1] Cozzani E., Drosera M., Gasparini G., Parodi A. (2014). Serology of lupus erythematosus: correlation between immunopathological features and clinical aspects. *Autoimmune Diseases*.

[2] Yap D. Y. H., Lai K. N. (2015). Pathogenesis of renal disease in Systemic Lupus Erythematosus—the role of autoantibodies and lymphocytes subset abnormalities. *International Journal of Molecular Sciences*.

[3] Moroni G., Quaglini S., Radice A. (2015). The value of a panel of autoantibodies for predicting the activity of lupus nephritis at time of renal biopsy. *Journal of Immunology Research*.

[4] Yang X.-W., Tan Y., Yu F., Zhao M.-H. (2012). Combination of anti-C1q and anti-dsDNA antibodies is associated with higher renal disease activity and predicts renal prognosis of patients with lupus nephritis. *Nephrology Dialysis Transplantation*.

[5] Yin Y., Wu X., Shan G., Zhang X. (2012). Diagnostic value of serum anti-C1q antibodies in patients with lupus nephritis: a meta-analysis. *Lupus*.

[6] Chi S., Yu Y., Shi J. (2015). Antibodies against C1q are a valuable serological marker for identification of systemic lupus erythematosus patients with active lupus nephritis. *Disease Markers*.

[7] Chi S., Xue J., Li F. (2016). Correlation of serum soluble interleukin-7 receptor and anti-C1q antibody in patients with systemic lupus erythematosus. *Autoimmune Diseases*.

[8] Mollnes T. E., Haga H.-J., Brun J. G. (1999). Complement activation in patients with systemic lupus erythematosus without nephritis. *Rheumatology*.

[9] Rovin B. H., Birmingham D. J., Nagaraja H. N., Yu C. Y., Hebert L. A. (2007). Biomarker discovery in human SLE nephritis. *Bulletin of the NYU Hospital for Joint Diseases*.

[10] Marto N., Bertolaccini M. L., Calabuig E., Hughes G. R. V., Khamashta M. A. (2005). Anti-C1q antibodies in nephritis: correlation between titres and renal disease activity and positive predictive value in systemic lupus erythematosus. *Annals of the Rheumatic Diseases*.

[11] Nusse R. (2015). Cell signalling: disarming Wnt. *Nature*.

[12] Hauck S. M., Hofmaier F., Dietter J. (2012). Label-free LC-MSMS analysis of vitreous from autoimmune uveitis reveals a significant decrease in secreted Wnt signalling inhibitors DKK3 and SFRP2. *Journal of Proteomics*.

[13] Tveita A. A., Rekvig O. P. (2011). Alterations in Wnt pathway activity in mouse serum and kidneys during lupus development. *Arthritis and Rheumatism*.

[14] Moon R. T., Kohn A. D., De Ferrari G. V., Kaykas A. (2004). WNT and *β*-catenin signalling: diseases and therapies. *Nature Reviews Genetics*.

[15] Wang X.-D., Huang X.-F., Yan Q.-R., Bao C.-D. (2014). Aberrant activation of the WNT/*β*-catenin signaling pathway in lupus nephritis. *PLoS ONE*.

[16] Shi J., Chi S., Xue J., Yang J., Feng L., Liu X. (2016). Emerging role and therapeutic implication of wnt signaling pathways in autoimmune diseases. *Journal of Immunology Research*.

[17] Xie W., Zhou L., Li S., Hui T., Chen D. (2016). Wnt/*β*-catenin signaling plays a key role in the development of spondyloarthritis. *Annals of the New York Academy of Sciences*.

[18] Olferiev M., Jacek E., Kirou K. A., Crow M. K. (2016). Novel molecular signatures in mononuclear cell populations from patients with systemic lupus erythematosus. *Clinical Immunology*.

[19] Tamai K., Semenov M., Kato Y. (2000). LDL-receptor-related proteins in Wnt signal transduction. *Nature*.

[20] Malinauskas T., Jones E. Y. (2014). Extracellular modulators of Wnt signalling. *Current Opinion in Structural Biology*.

[21] Niehrs C. (2006). Function and biological roles of the Dickkopf family of Wnt modulators. *Oncogene*.

[22] Mao B., Niehrs C. (2003). Kremen2 modulates Dickkopf2 activity during Wnt/LRP6 signaling. *Gene*.

[23] Liang B., Zhong L., He Q. (2015). Serum dickkopf-1 as a biomarker in screening gastrointestinal cancers: a systematic review and meta-analysis. *OncoTargets and Therapy*.

[24] Rossini M., Viapiana O., Adami S. (2015). In patients with rheumatoid arthritis, Dickkopf-1 serum levels are correlated with parathyroid hormone, bone erosions and bone mineral density. *Clinical and Experimental Rheumatology*.

[25] Miceli-Richard C., Taylor K. E., Nititham J. (2015). Genetic contribution of DKK-1 polymorphisms to RA structural severity and DKK-1 level of expression. *Annals of the Rheumatic Diseases*.

[26] Choe J.-Y., Kim J. H., Park K.-Y., Choi C.-H., Kim S.-K. (2016). Activation of dickkopf-1 and focal adhesion kinase pathway by tumour necrosis factor *α* induces enhanced migration of fibroblast-like synoviocytes in rheumatoid arthritis. *Rheumatology (United Kingdom)*.

[27] Juarez M., McGettrick H. M., Scheel-Toellner D. (2016). DKK1 expression by synovial fibroblasts in very early rheumatoid arthritis associates with lymphocyte adhesion in an in vitro flow co-culture system. *Arthritis Research and Therapy*.

[28] Deng Y. J., Huang Z. X., Zhou C. J. (2006). Gene profiling involved in immature CD4+ T lymphocyte responsible for systemic lupus erythematosus. *Molecular Immunology*.

[29] Ma J., Wang R., Fang X., Sun Z. (2012). *β*-catenin/TCF-1 pathway in T cell development and differentiation. *Journal of Neuroimmune Pharmacology*.

[30] Long L., Liu Y., Wang S. (2010). Dickkopf-1 as potential biomarker to evaluate bone erosion in systemic lupus erythematosus. *Journal of Clinical Immunology*.

[31] Orme J. J., Du Y., Vanarsa K. (2016). Leukocyte beta-catenin expression is disturbed in systemic lupus erythematosus. *PLoS ONE*.

[32] Jiang X.-T., Ma Y.-Y., Guo K. (2014). Assessing the diagnostic value of serum dickkopf-related protein 1 levels in cancer detection: A Case-control Study and Meta-analysis. *Asian Pacific Journal of Cancer Prevention*.

[33] Dong L.-L., Qu L.-Y., Chu L.-Y., Zhang X.-H., Liu Y.-H. (2014). Serum level of DKK-1 and its prognostic potential in non-small cell lung cancer. *Diagnostic Pathology*.

[34] Xu H., Wu J., Chen B. (2014). Serum Dickkopf-1 (DKK1) is significantly lower in patients with lung cancer but is rapidly normalized after treatment. *American Journal of Translational Research*.

[35] Han S.-X., Zhou X., Sui X. (2015). Serum dickkopf-1 is a novel serological biomarker for the diagnosis and prognosis of pancreatic cancer. *Oncotarget*.

[36] Jeng J.-E., Chuang L.-Y., Chuang W.-L., Tsai J.-F. (2012). Serum Dickkopf-1 as a biomarker for the diagnosis of hepatocellular carcinoma. *Chinese Clinical Oncology*.

[37] Shen Q., Fan J., Yang X. (2012). Serum DKK1 as a protein biomarker for the diagnosis of hepatocellular carcinoma: A Large-scale, Multicentre Study. *The Lancet Oncology*.

[38] Zhang J., Zhao Y., Yang Q. (2014). Sensitivity and specificity of Dickkopf-1 protein in serum for diagnosing hepatocellular carcinoma: a meta-analysis. *International Journal of Biological Markers*.

[39] Ustun N., Tok F., Kalyoncu U. (2014). Sclerostin and Dkk-1 in patients with ankylosing spondylitis. *Acta Reumatologica Portuguesa*.

[40] Yucong Z., Lu L., Shengfa L., Liang Y. Y., Ruguo S., Yikai L. (2014). Serum functional dickkopf-1 levels are inversely correlated with radiographic severity of ankylosing spondylitis. *Clinical Laboratory*.

[41] Tan E. M., Cohen A. S., Fries J. F. (1982). The 1982 revised criteria for the classification of systemic lupus erythrematosus. *Arthritis and Rheumatism*.

[42] Petri M., Kasitanon N., Lee S.-S. (2008). Systemic lupus international collaborating clinics renal activity/response exercise: development of a renal activity score and renal response index. *Arthritis and Rheumatism*.

[43] Gladman D. D., Ibañez D., Urowltz M. B. (2002). Systemic lupus erythematosus disease activity index 2000. *Journal of Rheumatology*.

[44] Touma Z., Gladman D. D., MacKinnon A. (2013). Development and assessment of users' satisfaction with the systemic lupus erythematosus disease activity index 2000 responder index-50 website. *Journal of Rheumatology*.

[45] Parikh R., Mathai A., Parikh S., Sekhar G. C., Thomas R. (2008). Understanding and using sensitivity, specificity and predictive values. *Indian Journal of Ophthalmology*.

[46] Benzing T., Simons M., Walz G. (2007). Wnt signaling in polycystic kidney disease. *Journal of the American Society of Nephrology*.

[47] Logan C. Y., Nusse R. (2004). The Wnt signaling pathway in development and disease. *Annual Review of Cell and Developmental Biology*.

[48] Pishvaian M. J., Byers S. W. (2007). Biomarkers of WNT signaling. *Cancer Biomarkers*.

[49] Madan B., Ke Z., Lei Z. D. (2016). NOTUM is a potential pharmacodynamic biomarker of wnt pathway inhibition. *Oncotarget*.

[50] Liu Y., Zhou R., Yuan X. (2015). DACH1 is a novel predictive and prognostic biomarker in hepatocellular carcinoma as a negative regulator of Wnt/*β*-catenin signaling. *Oncotarget*.

[51] Jiang L., Yang Y.-D., Fu L. (2014). CLDN3 inhibits cancer aggressiveness via Wnt-EMT signaling and is a potential prognostic biomarker for hepatocellular carcinoma. *Oncotarget*.

[52] Lee H.-H., Uen Y.-H., Tian Y.-F. (2009). Wnt-1 protein as a prognostic biomarker for hepatitis B-related and hepatitis C-related hepatocellular carcinoma after surgery. *Cancer Epidemiology Biomarkers and Prevention*.

[53] Urakami S., Shiina H., Enokida H. (2006). Combination analysis of hypermethylated Wnt-antagonist family genes as a novel epigenetic biomarker panel for bladder cancer detection. *Clinical Cancer Research*.

[54] Kim S. U., Park J. H., Kim H.-S. (2015). Serum dickkopf-1 as a biomarker for the diagnosis of hepatocellular carcinoma. *Yonsei Medical Journal*.

[55] Liu Q.-R., Li Y.-F., Deng Z.-Q., Cao J.-Q. (2016). Prognostic significance of Dickkopf-1 in gastric cancer survival: a meta-analysis. *Genetic Testing and Molecular Biomarkers*.

[56] Zhang L., Ouyang H., Xie Z., Liang Z., Wu X. (2016). Serum DKK-1 level in the development of ankylosing spondylitis and rheumatic arthritis: a meta-analysis. *Experimental & Molecular Medicine*.

[57] Daoussis D., Andonopoulos A. P. (2011). The emerging role of dickkopf-1 in bone biology: is it the main switch controlling bone and joint remodeling?. *Seminars in Arthritis and Rheumatism*.

[58] Miao C.-G., Yang Y.-Y., He X. (2013). Wnt signaling pathway in rheumatoid arthritis, with special emphasis on the different roles in synovial inflammation and bone remodeling. *Cellular Signalling*.

[59] Pinzone J. J., Hall B. M., Thudi N. K. (2009). The role of Dickkopf-1 in bone development, homeostasis, and disease. *Blood*.

[60] Kwon S.-R., Lim M.-J., Suh C.-H. (2012). Dickkopf-1 level is lower in patients with ankylosing spondylitis than in healthy people and is not influenced by anti-tumor necrosis factor therapy. *Rheumatology International*.

[61] Daoussis D., Liossis S.-N. C., Solomou E. E. (2010). Evidence that Dkk-1 is dysfunctional in ankylosing spondylitis. *Arthritis and Rheumatism*.

[62] Diarra D., Stolina M., Polzer K. (2007). Dickkopf-1 is a master regulator of joint remodeling. *Nature Medicine*.

[63] Uderhardt S., Diarra D., Katzenbeisser J. (2010). Blockade of Dickkopf (DKK)-1 induces fusion of sacroiliac joints. *Annals of the Rheumatic Diseases*.

